# Retraction: Application of Robot Positioning for Cannulated Screw Internal Fixation in the Treatment of Femoral Neck Fracture: Retrospective Study

**DOI:** 10.2196/102057

**Published:** 2026-06-16

**Authors:** JMIR Publications Editorial Office

**Affiliations:** 1 JMIR Publications Toronto, ON

The JMIR Publications Editorial Office is retracting the article “Application of Robot Positioning for Cannulated Screw Internal Fixation in the Treatment of Femoral Neck Fracture: Retrospective Study” [1].

This action is being taken due to identified concerns regarding potential manipulation of the submission process and the integrity of the peer-review process.

The authors were contacted and offered an opportunity to comment on these findings and the proposed retraction but did not respond to any attempts at communication.
